# Biochemical characterization and peptide mass fingerprinting of two glutathione transferases from *Biomphalaria alexandrina* snails (Gastropoda: Planorbidae)

**DOI:** 10.1186/s43141-022-00372-x

**Published:** 2022-07-06

**Authors:** Abdel-Monem Abdalla, Ghada S. A. Abdel Karim

**Affiliations:** Molecular Biology Department, Biotechnology Research Institute, National Research Centre, 33 El Bohouth St., Dokki, P.O. Box: 12622, Giza, Egypt

**Keywords:** *Biomphalaria alexandrina*, Glutathione transferase, Inhibition, Purification, Characterization, Enzyme-linked immunosorbent assay, Chromatography

## Abstract

**Background:**

The freshwater snails *Biomphalaria alexandrina* (Gastropoda: Planorbidae) has public health importance of being an intermediate host of *Schistosoma mansoni*, the parasite species that causes intestinal schistosomiasis in humans. Glutathione transferases (GSTs) play an important role in detoxification of a broad range of compounds including secondary metabolites and exogenous compounds. Studying GSTs in snails may clarify their role in detoxification of molluscicides.

**Results:**

Two glutathione transferases (BaGST2 and BaGST3) were purified and characterized from *B. alexandrina* snails. BaGST2 and BaGST3 were electrophoretically homogeneous preparations with subunit molecular weight of 23.6 kDa and molecular weight of 45 kDa. Isoelectric focusing of BaGST2 revealed the presence of two components at pI 4.47 and 4.67, while BaGST3 showed one band at pI 4.17. The specific activity of BaGST2 and BaGST3 toward 1-chloro-2,4-dinitrobenzene (CDNB) was 19.0 and 45.2 μmol/min/mg protein following 146- and 346-fold purification, respectively. The catalytic pH optima, km values, and the activation energies for BaGST2 and BaGST3 were determined. BaGST2 and BaGST3 were significantly inhibited by hematin and Cibacron Blue and to a less extent by bromosulfophthalein, S-butyl-GSH, S-hexyl-GSH, and S-P-bromobenzyl-GSH. BaGST2 and BaGST3 showed high activity against ethacrynic acid as substrate, and they also exhibited peroxidase activity on cumene hydroperoxide. The two enzymes showed identical patterns of lysine-C digestion after high-performance liquid chromatography. The amino acid sequences of three peptide fragments and peptide mass fingerprinting of fourteen peptides were used to predict the primary structure of BaGST2. A polypeptide of 206 amino acids (with 7 gaps, 3 of which could not identified) was predicted for BaGST2. The theoretical subunit molecular weight of BaGST2 is 22.6 kDa, with pI of 8.58. BaGST2 has 65% sequence identity and 78% positive with *Biomphalaria glabrata* GST7. The overall structure of BaGST2 at the N-terminal domain is identical to the canonical GST N-terminal domain, having the typical thioredoxin-like fold with a βαβ-α-ββα motif, whereas the C-terminal domain is made from 6 α-helices. A conservative GST-N-domain includes glutathione binding sites Y11, L17, Q53, M54, Q65, and S66, while a variable GST-C domain contains electrophilic substrate binding site H99, R102, A103, F106, K107, L161, and Y167. Phylogenetic tree showed that BaGST2 was clustered in the sigma group with GSTs sigma class from invertebrates and vertebrates.

**Conclusions:**

We have purified and characterized two GSTs from *B. alexandrina* snails. Our study broadens the biochemical information on freshwater snail GSTs by demonstrating the role of BaGSTs in defense mechanisms against structurally different electrophilic compounds. BaGST2 and BaGST3 have Se-independent peroxidase activity, which indicates their role in cellular antioxidant defense by reducing organic hydroperoxides in *B. alexandrina*. A polypeptide chain of 206 amino acids was predicted. The primary structure of BaGST2 showed 65% sequence identity with *Biomphalaria glabrata* GST7. Sequence analysis indicates that BaGST2 is a GST-N-sigma-like with a thioredoxin-like superfamily. Phylogenetic tree confirms that BaGST2 belongs to the sigma class of GSTs superfamily.

**Supplementary Information:**

The online version contains supplementary material available at 10.1186/s43141-022-00372-x.

## Background

Glutathione transferases (GSTs, EC 2.5.1.18) have a widespread distribution in all living organisms. They make up a superfamily of enzymes with an important role as phase ІІ enzymes in the cellular defense against different electrophilic compounds. GSTs catalyze the conjugation of substances that have an electrophilic carbon, nitrogen, or sulfur atom to reduced glutathione (γ-L-glutamyl-L-cysteinyl-glycine, GSH). GSTs contribute to the detoxification of drugs, pesticides, molluscicides, and other foreign compound. Some products of oxidative stress, such as epoxides, hydroperoxides, and alkenes, are also substrates [1–3]. Based on the homology of amino acid sequences, cytosolic GSTs are grouped into many distinct classes, namely alpha, mu, pi, theta, sigma, omega, zeta, delta, epsilon, lambda, tau, phi and beta, xi, chi, iota, and rho [4–6]. A typical characteristic of all cytosolic GSTs is their existence as homo- or heterodimers having two substrate binding sites in each subunit: a highly specific and conserved glutathione binding site (G-site) and a hydrophobic binding site (H-site) for the electrophilic substrates. However, lambda GST from *Populous trichocarpa* is reportedly a strictly monomeric protein [7].

Despite the medical importance of freshwater snails as intermediate hosts of *Schistosoma* parasites, only few GSTs have been characterized [8]. The snail *B. alexandrina* (Gastropoda: Planorbidae) is a freshwater snail of public health importance being an intermediate host of *S. mansoni*, the parasite species that causes intestinal schistosomiasis in humans. Schistosomiasis is one of the major communicable diseases in the developing world and a public health issue of significant socio-economic importance. Despite control efforts in a number of countries, an estimated 200 million people are infected, of which 120 million are symptomatic and 20 million have severe debilitating disease [9, 10].

Treatments of habitats with molluscicides reduce the size of snail populations, but it has not led to their elimination. To avoid or reduce potential toxic insult, snails present behavioral and physiological mechanisms, which may differ among species. Comparing with vertebrates, there is a little information available concerning GSTs of aquatic invertebrates, such as freshwater snails.

Drug-metabolizing enzymes belonging to the GST superfamily are multifunctional phase II proteins primarily involved in the cellular detoxification of both endogenous and exogenous compounds [3]. GSTs in *B*. *alexandrina* snails could potentially be targeted for designing a specific inhibitor(s) to decrease molluscicide resistances. However, to the best of our knowledge, no literature is available to date on *B. alexandrina* GSTs with regard to GST classes and structural information. Thus, this study is aimed at addressing this research gap. In this study, purification, characterization, peptide mass fingerprinting, and structural analysis of GSTs in *B. alexandrina* species have been carried out.

## Methods

### Materials

*B. alexandrina* snails were obtained from Abu-Rawah area, Giza Governorate, Egypt. Snails with approximately 10 ± 2 mm were selected, maintained in laboratory under standard conditions of aeration and temperature range 25–30 °C. They were fed with fresh lettuce leaves and placed in dechlorinated water several days prior to being used in the experiment. The snails were then collected, washed with tape water, and saved at −20. Glutathione (GSH) and 1-chloro-2,4-dinitrobenzene (CDNB) were purchased from Merck. Sephadex G-75, epoxy-activated Sepharose 6B, and molecular weight standard proteins were purchased from the Amersham-Pharmacia Biotechnology. Other chemicals were of the highest purity commercially available.

#### Preparation of GSH-Sepharose affinity matrix

Glutathione was coupled to epoxy-activated Sepharose 6B according to Simons and Vander Jagt [11].

##### Glutathione transferase assay

The GST activity was determined spectrophotometrically at 25 °C with GSH and CDNB as substrates by monitoring the change in absorbance due to thioether formation at 340 nm as previously described [12]. One unit of transferase activity is defined as the amount of enzyme which catalyzes the formation of 1 *μ*mol of thioether per min, and the specific activity is expressed as *μ*mol/min/mg protein.

##### Protein determination

Protein was determined according to the method of Bradford [13] with bovine serum albumin as a standard.

### Purification of GSTs from B. alexandrina

#### Enzyme extraction

The known weight of the whole animal was homogenized in 50 mM Tris-HCl buffer, pH 8.0 containing 2.0 mM dithiothreitol, and 2 mM ethylenediaminetetraacetic acid (EDTA) using Omni mixer. The homogenate was then centrifuged at 33,000 × g for 1 h. The supernatant was filtered through a plug of glass wool to remove floating lipids. The filtrate was termed crude extract.

#### DEAE-cellulose column chromatography

The dialyzed fraction was applied on a DEAE-cellulose column (1.6 × 25 cm i.d.) previously equilibrated with 25 mM Tris-HCl buffer, pH 8.0 (buffer A). After sample application, the column was washed with the same buffer, and the adsorbed proteins were eluted using stepwise NaCl gradient ranging from 50 to 500 mM in equilibration buffer. A total of 10-mL fractions were collected at a flow rate of 60 mL/h. The eluted fractions were monitored at 280 nm and assayed for enzyme activity. Fractions containing enzyme activity were found in four major separate peaks that were separately pooled.

#### Affinity column chromatography of GST

The sample to be affinity purified was passed through GSH-Sepharose column (1 × 15 cm i.d.) previously equilibrated with buffer A at a flow rate of 15 mL/h. Unbound material was washed from the column at a flow rate of 30 mL/h with 150 mM NaCl in the equilibration buffer until the absorbance at 280 nm reached zero. The enzyme was eluted with 10 mM GSH in 50 mM Tris-HCl buffer, pH 9.6 at a flow rate of 15 mL/h. A total of 2-mL fractions were collected and monitored for protein at 280 nm and for glutathione transferase activity.

#### Polyacrylamide gel electrophoresis (PAGE)

Native PAGE was carried out according to Davis [14]. The gel was stained for GST activity according to the method described by Ricci [15] and for protein with Coomassie Brilliant Blue R-250. The subunit molecular weight (MW) of the protein was determined by SDS-PAGE according to the method described by Laemmli [16].

### Molecular mass determination

The apparent MW was determined by size exclusion chromatography on a column (90 ×1.4 cm i.d.) packed with Sephadex G-75, equilibrated with 20 mM Tris-HCl buffer, pH 8.0 containing 100 mM NaCl at 4 °C. The column was calibrated with the following molecular standard proteins: albumin (67 kDa), ovalbumin (43 kDa), chymotrypsinogen A (25 kDa), and ribonuclease A (13.7 kDa). Ten units of pure GST enzyme and 2 mg of the standard proteins were applied to the column at a flow rate of 6 mL/h, and 2-mL fractions were collected and monitored by absorbance at 280 nm for the standard proteins or by GST activity.

### Electrofocusing

The isoelectric point (pI) was determined by comparing the mobility of the tested protein in a stable pH gradient with standard proteins of known pI using 5.5% polyacrylamide gel containing 3% ampholine (pH 3–10) at 4 °C in a horizontal electrophoresis system (Amersham Pharmacia Biotech).

### Glutathione transferase assay using other substrates

Enzyme activity with various aromatic substrates, namely, bromosulfophthalein (BSP), 1, 2-dichloro-4-nitrobenzene (DCNB), p-nitrophenethyl-bromide, and ethacrynic acid (EA), was examined as described [12]. GSTs peroxidase activity with cumene hydroperoxide (CuOOH) as substrate was measured according to Weinhold et al. [17].

### Effect of inhibitors and type of inhibition

Cibacron Blue (CB), BSP, hematin, *S*-butyl-GSH, S-hexyl-GSH, and *S*-P-bromo-benzyl-GSH were tested for their ability to inhibit GSH-CDNB-conjugating activity of the purified BaGST2 and BaGST3. The IC_50_ values were determined according to Yalcin [18]. The IC_50_ values were determined by plotting percentage activity values versus log inhibitor concentrations.

### Thermal stability

The thermal stability of enzyme was measure as a function of time. The enzyme (25 μg/ml) was incubated in 100 mM phosphate buffer, pH 7.0 containing 1 mM EDTA and 1 mM DTT at 50 °C in the absence or presence of 5 mM GSH. Aliquots were assayed for GST conjugated activity at different timepoints. The half-life of BaGST2 or BaGST3 represents the time of incubation when there is 50% residual activity.

### Preparation of rabbit antisera against BaGST2 and BaGST3

Two young female New Zealand rabbits (1.5 kg) each received two injections of a total of 100 μg of electrophoretically pure BaGST2 or BaGST3. The initial injection, 60 μg antigen in phosphate-buffered saline (PBS) emulsified in an equal volume of Freund’s complete adjuvant, was administered subcutaneously. After 21 days, another subcutaneous injection of 40 μg of the same antigen was used to boost the immune response. Fifteen days after the booster injection, blood samples were drawn from the central artery, allowed to clot, and centrifuged, and the serum was collected. Serum was stored frozen in aliquots at −20 °C.

### Enzyme-linked immunosorbent assay (ELISA)

A micro-titer plate was coated with the protein fractions to be analyzed. Twofold serial dilutions of protein were made in 0.1 M carbonate buffer, pH 9.5, and the protein was allowed to bind. After washing with phosphate-buffered saline/Tween 20 (PBS/Tw), the plates were blocked with 2% BSA in PBS and washed as above. Twofold serial dilutions of antisera in PBS/Tw containing 2% BSA were added, followed by adding conjugated goat peroxidase with anti-rabbit IgG followed by washing. Finally, 50 μl of peroxidase substrate (0.04% *O*-phenylenediamine dihydrochloride and 0.012% H_2_O_2_ in 100 mM citric acid-sodium phosphate buffer, pH 5.0) was added, and after 30 min of incubation, the reaction was stopped with 50 μl of 2 M HCl, and the absorbance at 490 nm was determined using a micro-titer plate reader.

### Amino acid composition

The sample to be analyzed was first hydrolyzed to free amino acid with 6 N HCl. After evaporation of HCl, the hydrolysate was dissolved in appropriate volume of the application buffer and applied to the amino acid analyzer. Norleucine (10 nmol) was used as internal standard.

### Peptides separation and amino acid sequence

In situ digestion, peptides separation, and sequence determination were done as described [19]. A total of 40 μg of the pure BaGST2 and BaGST3 protein was first reduced with 10 mM DTT at 95 °C and then alkylated in the dark with 20 mM iodoacetamide. The reduced samples were subjected to one-dimensional SDS-PAGE. After staining and destaining, the bands were excised and treated for in situ digestion. Digestion was carried out with lysine-C-specific protease. Reversed-phase liquid chromatography (HPLC) was used to separate the fragments using a gradient of acetonitrile in TFA. The amino acid sequences were determined using a gas-phase sequencer (Applied Biosystem, model 477A) fitted with an online PTH-derivative analyzer (model 120A).

### Sequence analysis and bioinformatics procedures

GSTs sequences were downloaded from the UniProt Knowledgebase [20]. The theoretical digestion of GSTs sequences, and prediction of molecular weight and isoelectric point (pI), was carried out using (https://web.expasy.org/peptide_mass/) [21]. Conserved domains were identified by the tool of the CD-search based on the Conserved Domain Database (CDD) (https://www.ncbi.nlm.nih.gov/Structure/cdd/wrpsb.cgi) [22]. Secondary structure was identified using the protein secondary structure prediction server, JPred4 (http://www.compbio.dundee.ac.uk/jpred4/index_up.html) [23]. Sequences alignment and phylogenetic tree were done using Clustal Omega (https://www.ebi.ac.uk/Tools/msa/clustalo/) [24].

## Results

### Purification, homogeneity, subunit molecular weight, and isoelectric point

**Fig. 1:**
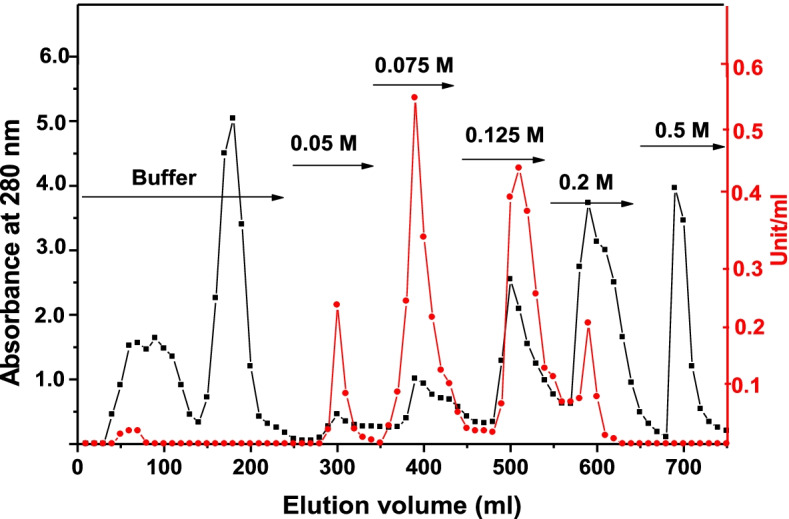
Elution profile for chromatography of *B. alexandrina* crude extract on DEAE-cellulose (DE-53) column. Absorbance at 280 nm (-■-) and GST activity measured using CDNB

**Fig. 2:**
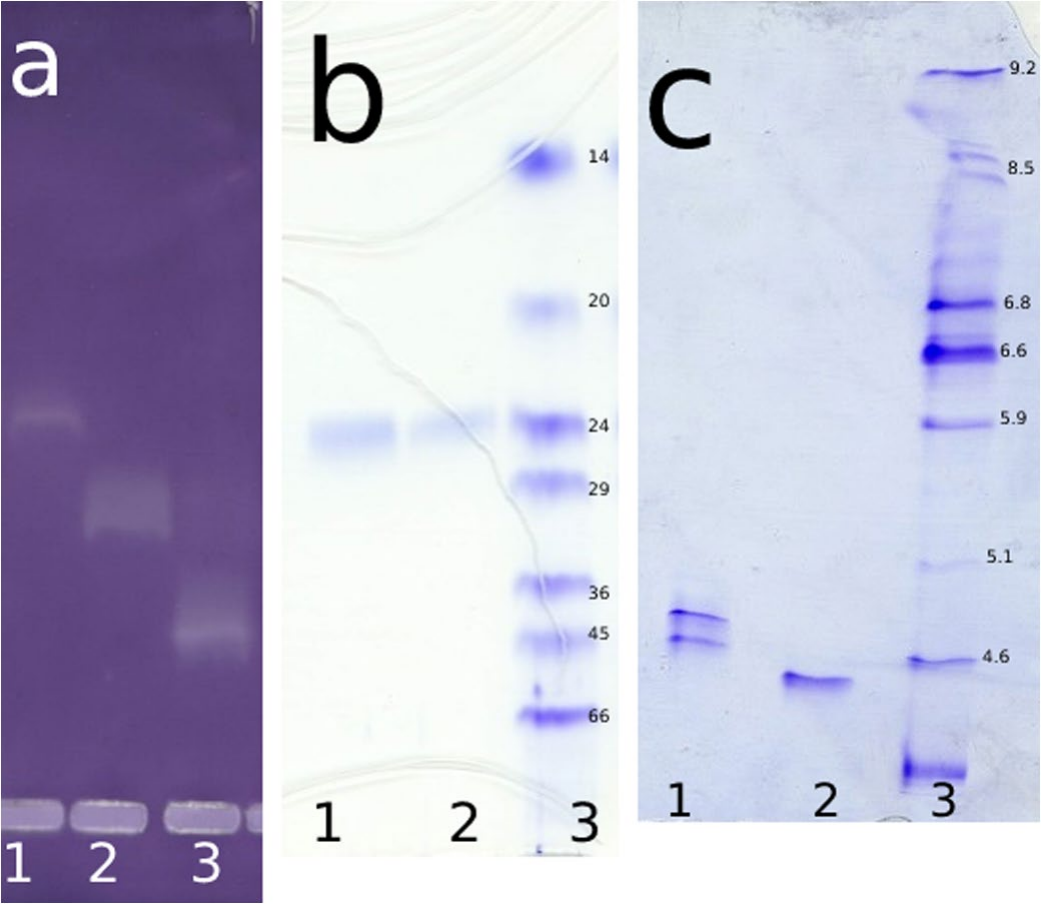
**a** Native PAGE of *B. alexandrina* GSTs stained for (lane1: BaGST1, lane 2: BaGST2, and lane 3: BaGST3). **b** SDS-PAGE of BaGST2 and BaGST3 stained for protein (lane 1: BaGST2, lane 2: BaGST3, and lane 3: Standard molecular weight markers). **c** foucusing of BaGST2 and BaGST3 (lane 1: BaGST2, lane 2: BaGST3, and lane 3: Standard isoelectric focusing markers)

The results of the purification of the two major GST enzymes present in *B. alexandrina* are summarized in Table 1. GSH-conjugating activity toward CDNB in the crude extract was 0.13 μmol/min/mg protein. Following chromatography on DEAE cellulose of the crude extract, five peaks of GST activity (including the flow-through) were eluted between 0 and 200 mM NaCl with a recovery of 81.6% (Fig. 1). After passage of the two major fractions through the GSH Sepharose column, the specific activity of BaGST2 and BaGST3 was 19.0 and 45.2 μmol/min/mg protein, respectively, with an overall recovery of 49.6% of the initial activity (Table 1). The two GST enzymes gave a single band on native PAGE stained for activity (Fig. 2a). Comparison of relative mobility of BaGST2 and BaGST3 with standard proteins indicated a MW of approximately 23.6 kDa by SDS-PAGE (Fig. 2b). Analytical isoelectric focusing of BaGST2 resulted in two equally stained bands at pI 4.47 and 4.67. BaGST3 showed one band at pI 4.17 (Fig. 2c).

**Table 1:** Purification of GSTs from *B. alexandrina* snails

Step	Activity (units)	Protein (mg)	Specific activity (units/mg protein)	Purification (fold)	Recovery (%)
Crude extract	54	420	0.13	1.0	100
DEAE cellulose					
BaGST_1_	4.65	10.6	0.44	3.35	8.6
BaGST2	17.0	32.53	0.52	4.02	31.5
BaGST3	22.4	88.52	0.25	1.95	41.5
GSH-Sepharose					
BaGST1	2.93	0.13	22.5	170	5.4
BaGST2	10.09	0.53	19.0	146	20.2
BaGST3	16.73	0.37	45.2	346	31.0

### Kinetic studies

The response of the purified BaGST2 and BaGST3 to the variation of GSH and CDNB concentrations was studied at 25 °C. Km and V_max_ values were calculated and given in Table 2. The effect of CDNB concentrations on GSTs activities was examined between 0.01 and 1.5 mM under the standard assay condition of GST. Both isoenzymes exhibited typical Michaelian behavior in these ranges of substrate concentrations. The apparent affinity of BaGST3 to CDNB (0.05 mM) is almost sixty times higher than that of BaGST2 (3.33 mM). The effect of GSH concentrations on GSTs activities was examined in the range between 0.025 and 1.5 mM under the standard assay condition of GST. BaGST2 exhibited typical Michaelian behavior, and the Km value was calculated to be 0.22 mM. BaGST3 exhibited non-Michaelian behavior (concave downward) where at low GSH concentrations ranging between 0.025 and 0.5 mM, the Km was 0.15 mM, and at high GSH concentrations ranging from 0.5 to 5 mM, the Km was 0.555 mM.

**Table 2:** Substrate specificities (μmol/min/mg) of BaGST2 and BaGST3 toward some of GST substrates

Substrate	DCNB × 10^3^	EA	BSP	p-Nitro-phenethylbromide	CuOOH
BaGST2	30 ± 1.5	3.80 ± 0.11	ND	2.70 ± 0.13	0.60 ± 0.02
BaGST3	10 ± 0.6	2.60 ± 0.10	ND	6.00 ± 0.20	1.40 ± 0.03

*ND* no detected activity. Values are means ± SD, generally based on *n* ≥ 4

### Optimum pH, activation energy, and thermal stability

The effect of pH on BaGST2 and BaGST3 activities was examined between pH 3.5–9.5 using 0.1 M acetate buffer (pH 3.5 to 5.5) and 0.1 M phosphate buffer (pH 5.5 to 8.0) and 100 M Tris-HCl buffer (pH 8.5 to 9.5). As shown in Table 2, BaGST3 exhibited an acidic pH optimum at pH 6.0, whereas BaGST2 exhibited an alkaline pH optimum above pH 9.5. The effect of temperature on CDNB conjugation reaction catalyzed by BaGSTs was examined under the standard assay conditions. The reaction was carried out at temperatures ranging from 25 to 70 °C, and the activity of the enzyme was measured. The activity of BaGST2 and BaGST3 increased by increasing the temperature up to 60 °C and then started to decrease. The activation energy calculation from Arrhenius plot was 3.75 and 2.28 kcal/mol for BaGST2 and BaGST3, respectively (Table 2).

### Substrate specificity

Substrate specificities of BaGST2 and BaGST3 toward several compounds were studied. BaGST2 and BaGST3 did not show any detectable activity against BSP, showing a low activity toward DCNB and p-nitrophenthyl bromide. However, they showed high activity against EA as substrate. BaGST2 and BaGST3 exhibited also peroxidase activity on CuOOH (Table 2).

### Thermal stability

Thermal stability of the purified BaGST2 and BaGST3 at 50 °C was studied by monitoring the decrease in catalytic activity with time. As shown in Table 3, the half-life time of BaGST2 was 15 min, while that of BaGST3 was 25 min. Addition of 5 mM GSH increased the half-life of BaGST2 and BaGST3 to more than 180 min.

**Table 3:** Km, V_max_ (μmol/min/mg protein), pH optima, isoelectric point, IC_50_, activation energy, and half-life time at 50 °C of BaGST2 and BaGST3

Enzyme	Km (mM)		V_max_	pH optimum	pI	IC_50_ (μM)			Activation Energy (kcal/mol)	Half-life time at 50 °C	
	GSH	CDNB				S-butyl-GSH	S–hexyl-GSH	S-P-bromobenzyl-GSH		No GSH	+ 5 mM GSH
BaGST2	0.22	3.33	80	> 9.5	4.47 and 4.67	69.1	36.3	9.3	3.75	15 min	> 180 min
BaGST3	0.15 and 0.61	0.05	50	6.0	4.17	51.2	19.5	34.9	2.27	25 min	> 180 min

Values are means of at least four measurements

### Effect of inhibitors and type of inhibition

IC50 values determined under standard assay conditions spanned a range of three orders of magnitude (Tables 3 & 4). Hematin and CB were the most potent inhibitors, with IC_50_ values ranged between 0.46 and 2.35 μM, respectively, whereas IC_50_ of other compounds ranged between 9.3 and 95.0 μM (Table 4). The type of inhibition of BSP, hematin, and CB was also examined by studying the effect of constant concentration (less than IC_20_) of each inhibitor on the Km (CDNB) and Vmax of BaGST2 and BaGST3. As shown in Table 3, CB was found to be competitive inhibitor for BaGST2 and BaGST3. BSP was competitive for BaGST2 and mixed for BaGST3, whereas hematin was a mixed inhibitor for BaGST2 and noncompetitive inhibitor for BaGST3.

**Table 4:** IC_50_ (μM), Km (μM), Vmax (μmol/min/mg protein), and type of inhibition for BaGST2 and BaGST_3_ using CDNB as a substrate. The type of inhibition was measured in the presence of less than I_20_ μM of inhibitor

Inhibitor used	BaGST2				BaGST3			
	IC_50_	Km	V_max_	Type of inhibition	IC_50_	Km	V_max_	Type of inhibition
Non	-	0.85	75.5	-	-	50.0	48.8	-
BSP	80.4	2.75	75.5	Competitive	95.0	450	48.8	Mixed
Hematin	1.35	2.54	13.7	Mixed	0.46	50.0	15.8	Noncompetitive
CB	2.35	3.85	75.5	Competitive	0.58	750	48.8	Competitive

### Immunological characterization of BaGST2 and BaGST3

**Fig. 3:**
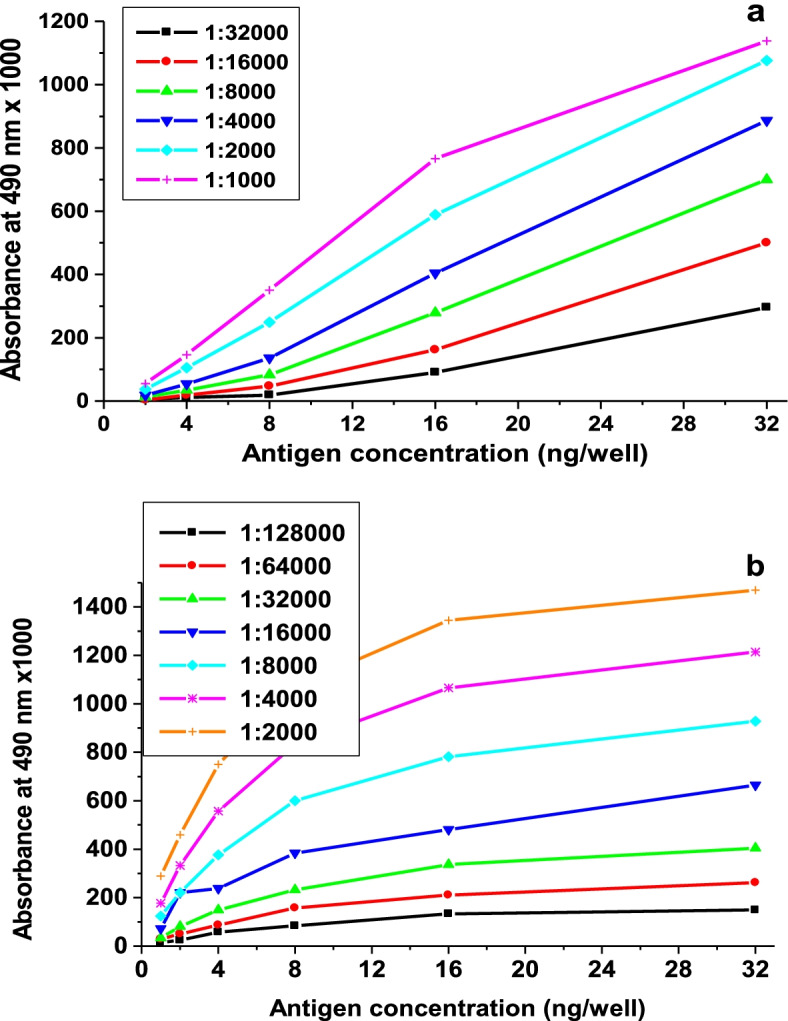
Determination of the optimal concentrations of the antigen and antibodies used in ELISA test. The micro-titer plate was sensitized with different concentrations of BaGST2 or BaGST3 starting from 640 ng/mL and ending with 20 ng/mL. The serial dilution of anti-BAGST2 polyclonal antibodies used was from 1:1000 to 1:32000 (**a**) and that for anti-BaGST3 was from 1:2000 to 1:128000 (**b**)

Polyclonal antibodies prepared against BaGST2 and BaGST3 were tested using ELISA technique. The optimal concentration of BaGST2 and BaGST3 and polyclonal antibodies were assessed by checkerboard (Fig. 3). Antibodies raised against BaGST2 did not cross-react with the BaGST1 and BaGST3 proteins. It also did not cross-react with the affinity-purified GSTs from *Schistosoma mansoni*, *Bulinus truncatus*, and rat liver. However, there is a low cross-reactivity with *Lymnaea truncatula* GSTs (less than 40 %) and strong cross-reactivity with *Physa acuta* GSTs. Anti-BaGST3 antibodies did not cross-react with BaGST1 and BaGST2. It also did not cross-react with GSTs from *S. mansoni*, *B. truncatus*, and rat liver. It showed low cross-reactivity with GSTs from *L. truncatula* and *P. acuta*.

### Amino acid composition, peptides separation, amino acid sequences, and peptide mass fingerprinting of BaGST2 and BaGST3

**Fig. 4:**
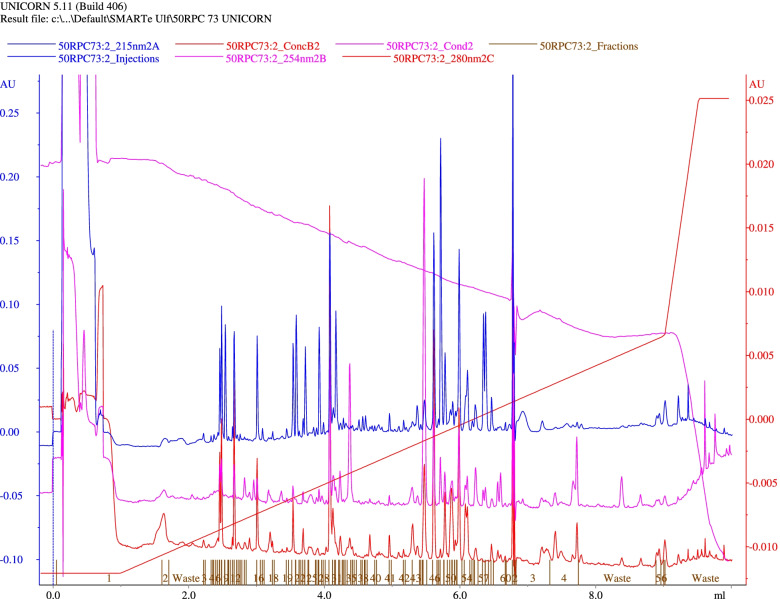
Reverse-phase high-performance liquid chromatography of lysine-C digest of BaGST2. A total of 40 μg of pure sample was reduced with 10 mM DTT at 95 °C, then alkylated in the dark with 20 mM iodoacetamide, and then subjected to SDS-PAGE. After staining and destaining, bands were excised and treated for in situ digestion with lysine-C-specific protease

Amino acid compositions of BaGST2 and BaGST3 are very similar. Cysteine and tryptophan could not be determined after acid hydrolysis (Supplementary Table S1). Reverse-phase high-performance liquid chromatography of a lysine-C digest of BaGST2 is represented in Fig. 4. Peptide patterns as well as peptide masses spectrometry of BaGST2 and BaGST3 indicate that both enzymes are very similar. Three peptides from BaGST2 were selected, and their amino acid sequences were determined. Peptide mass fingerprinting was used to get the amino acid sequences of other peptides. To predict the correct sequence, over 2000 GST sequences were downloaded from database and subjected to theoretical digestion using Lys-C peptidase. Peptides, whose masses matched with that measured by HPLC, were selected. The amino acid sequences of these peptides and that obtained by using the gas-phase sequencer are shown in Table 5.

**Table 5:** BaGST2 peptides masses measured by HPLC. Peptide masses and their amino acid sequences were obtained by theoretical Lys-C digestion of different GSTs. The amino acid sequences of peptide nos. 3, 9, and 15 were determined using a gas-phase sequencer. The values were rounded to the nearest tenths

Peptide no.	Peptide mass	NM	Position	Peptide sequence	GST source that match to BaGST2
1	890.4	1	1–8	MAEAKNVK	*Sphingomonas*
2	2465.5	1	9–30	VLYFDVTGLGEILRLLLKFAGK	*B. glabrata*
3	1859.9	0	31–44	EYEDVRFSFEEWPK	*B. glabrata*
4	1843.0	1	47–63	PTTPFGQMPVLEVDGKK	*Copidosoma floridanum*
5	1635.9	0	65–79	AQSIALAAFLAREFK	*B. glabrata*
6	2338.2	0	84–104	DDLEALQVDATVDTIHDLRAK	*Dufourea novaeangliae*
7	1065.6	0	108–115	SFRESDPVK	*B. glabrata*
8	1495.5	1	105–115	RFKSFRESDPVK	*B. glabrata*
9	888.6	2	116–123	EAIVTEVK	*B. glabrata*
10	1345.8	1	130–140	FMGFFESLLKK	*B. glabrata*
11	979.5	0	143–152	NGSTGLFVGK	*Haliotis discus discus*
12	1235.7	2	142–150	KNGSTGLFVGKK	*Haliotis discus*
13	1057.5	1	140–149	NGGHFVNGKK	*Ooceraea biroi*
14	1864.0	0	154–169	LTWGDFVFAGIYAYLK	*Epiphyas postvittana*
15	1434.8	0	170–182	AAFEAIDNFPLVK	*B. glabrata*
16	1358.7	0	183–194	LVDTVGDNERIK	*B. glabrata*
17	1234.6	1	195–206	WIETRPASKF	*Haemonchus contortus*

**Fig. 5:**
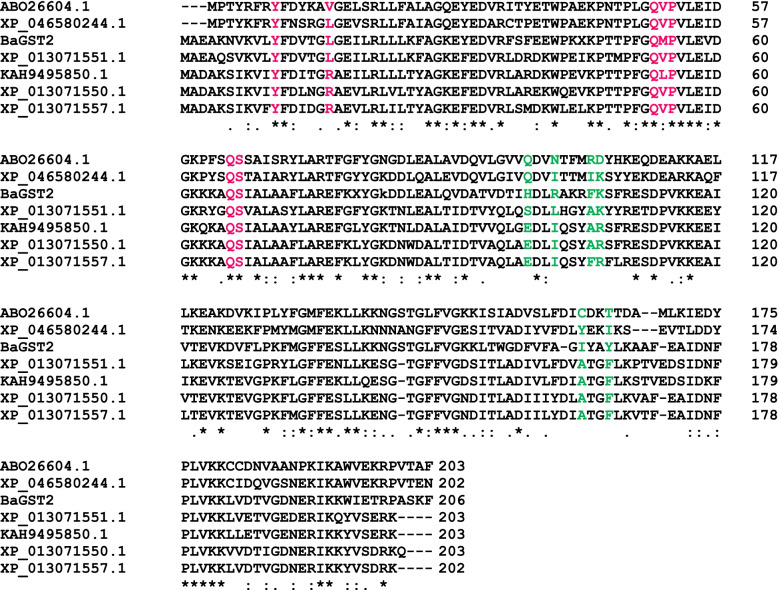
Multiple alignment of BaGST2 sequence with GST sequences from freshwater snails. The alignment was created using Clustal Omega multiple sequence alignment program. (*) indicates identical residues in all sequences, while (**:**) indicates the highly positive residues and (**.**) for moderately positive ones. Unidentified gaps marked by hyphens are introduced for better alignment. The conserved G-site residues are shown in red, and the substrate binding pockets (H-site) are shown in green

As shown in Table 5, peptide with a mass of 1065.6 Da (no. 7) is a product of zero missed cleavage of peptide no. 8 with a mass of 1495.5 Da. Peptide no. 13 cannot be fitted inside the predicted polypeptide chain unless it replaced peptide no. 11 (as they have the same position) (Fig. 5)*.* Only peptide no. 11 was incorporated in the alignment as it gives higher similarity than peptide no. 13, and it is also 0 missed cleavage.

### Sequence analysis

BaGST2 sequence with a polypeptide of 206 amino acids (including gaps), a MW of 22.6 kDa, and a pI of 8.58 was predicted. BaGST2 has the highest sequence identity with *B. glabrata* GST7 (65%) with 78% positive (accession no.: XP_013071550.1). The predicted polypeptide showed 60%, 59%, 48%, and 46% identity with *B. truncates* GST1(accession no.: KAH9495850.1), *B. glabrata* GST3 (accession no.: XP_013071551.1), *Haliotis discus discus* GST3 (accession no.: ABO26604.1), and *Haliotis rubra* GST2 (accession no.: XP_046580244.1), respectively. Multiple sequence alignment of BaGST2 sequence and that of GSTs from aforementioned freshwater snails species showed that there are 62 amino acids are identical. Furthermore, the amino acid-based alignment demonstrated that the N-terminal region appeared to be 40% conserved, while the C-terminal region was only 21% conserved (Fig. 5). Multiple alignment of BaGST2 with sigma-class GSTs of human (accession no.: O60760), rat (accession no.: O35543), and mouse (accession no.: Q9JHF7) showed that 56 amino acids are identical (Supplementary Fig. S2).

**Fig. 6:**
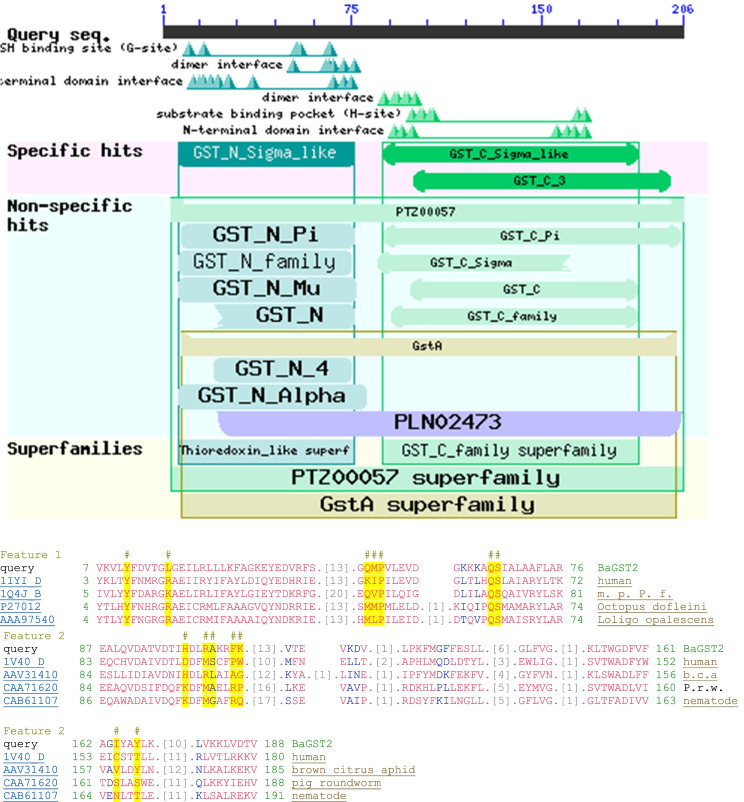
Conserved domains search of BaGST2. Conserved domains were identified by the tool of the CD-Search based on the conserved domain database (CDD) (https://www.ncbi.nlm.nih.gov/Structure/cdd/wrpsb.cgi). The G-site binding moieties are denoted by # in feature 1, and H-site binding moieties are denoted by # in feature 2

In silico secondary structure of BaGST2 was predicted by JPred4. The BaGST2 sequence was submitted to predict the closely related homologs. The structure of the *Bombyx mori* GST sigma (PDB ID: 3vpq) was selected as template for homology modeling (Blast *E*-value 1e-37). It predicted a structure with a classical GST-sigma βαβαββα motif in the N-terminal domain, and the C-terminal is composed of α-helices (Supplementary Fig. S3). The conserved domain search reveals that BaGST2 has two typical GST domains at the N- (5–82 amino acids) and C- (84–206 amino acids) termini, which match well with the coding domains of GST_N_Sigma_like (CDD accession no.: cd03039) and GST_C_Sigma_like (CDD accession no.: cd03192). These conserved domains are characteristic of sigma-class GSTs. The N-terminal domain, with a thioredoxin-like superfamily, includes GSH binding moieties Y11, L17, Q53, M54, P55, Q66, and S67, while that of GST-C domain contains substrate binding pocket H99, R102, A103, F106, K107, L161, and Y167 (Fig. 6). The details of dimer interface and domain interface residues in both the N-terminal and the C-terminal domain of BaGST2 are shown in Supplementary Fig. S4.

### Phylogenetic relationship and sigma GSTs homology analysis

**Fig. 7:**
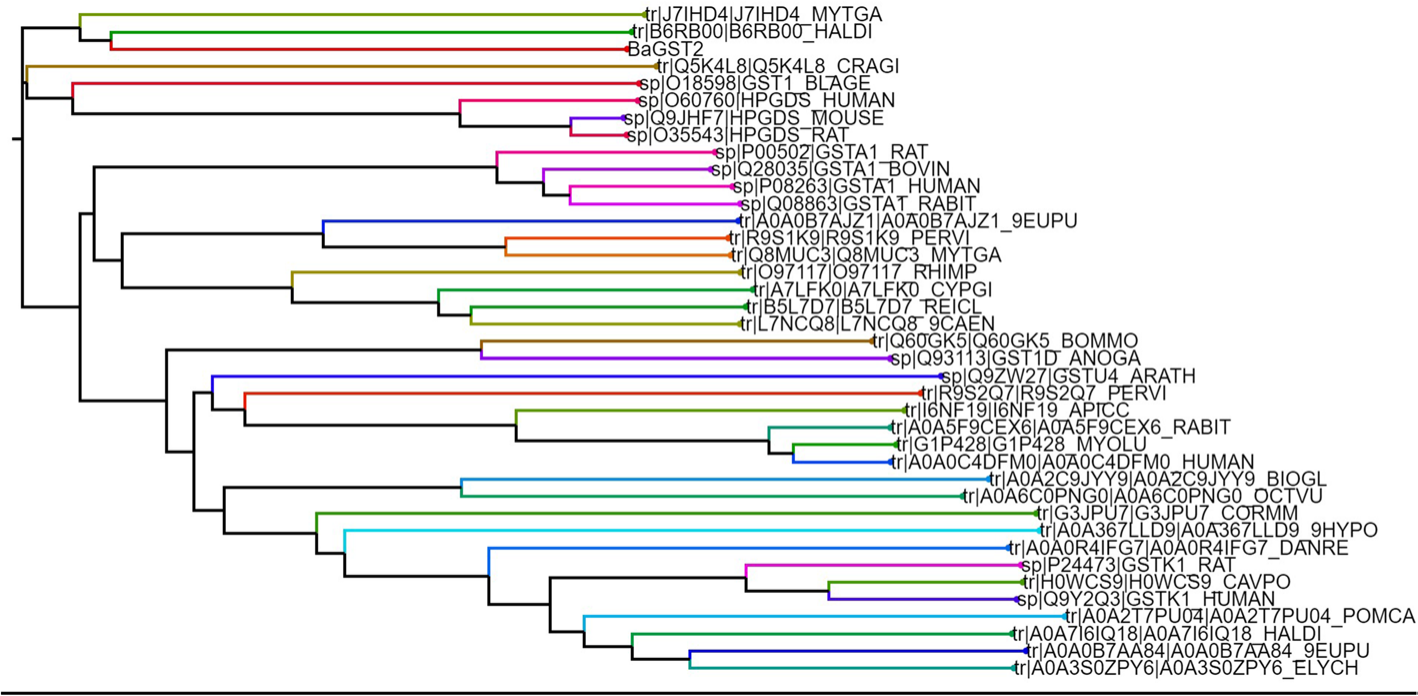
Phylogenetic relationships between BaGST2 and GSTs from different classes. BaGST2 sequence with GSTs from 41 different GST classes (downloaded from the UniProt Knowledgebase) was aligned first using Clustal Omega (https://www.ebi.ac.uk/Tools/msa/clustalo/), and then, the phylogenetic tree was reconstructed. Sigma-class GSTs are shown in yellow color

GST protein sequences of different classes in various species of invertebrates and vertebrates were downloaded from UniProt Knowledgebase [20], which were used to make a phylogenetic tree for inferring their evolutionary relationship. As shown in Fig. 7, the same class of GSTs clustered together in the tree. BaGST2 was obviously clustered in the sigma group and shared the evolutionary clade with sigma-class GSTs from human, rat, mouse, *Crassostrea gigas*, *Blattella germanica*, *Nototodarus sloanii*, *Mytilus galloprovincialis*, and *Haliotis discus discus*. However, BaGST2 is very distinct from invertebrates and vertebrates GSTs from other GST classes such as mu, alpha, pi, and kappa.

## Discussion

Chromatography of *B. alexandrina* snail homogenate on DEAE cellulose revealed the presence of three minor and two major GST isoenzymes. This result provides evidence for the existence of at least five separate forms of GST in *B. alexandrina*. In this respect, *B. alexandrina* is similar to other freshwater snails such as *P. acuta* and *B. truncates* and similar to other invertebrates or vertebrates [25–29]. BaGST2 and BaGST3 were purified by anion exchange and affinity chromatography to apparent homogeneity as judged by native and SDS-PAGE. The specific activities of the isolated BaGST2 and BaGST3 were 19.0 and 45.2 μmol/min/mg protein after 146 and 346-fold purification, respectively. The yield was 49.6% of the cytosolic GST activity. The purification yield is relatively low compared to that of other freshwater snail GSTs [25], but it is quite high if compared with purification of GSTs from insects [30, 31].

BaGST2 and BaGST3 were retained on GSH-affinity column, and their apparent values for GSH were calculated at 0.22 mM for BaGST2 and at 0.15 and 0.61 mM for BaGST3. These values are in general agreement with published Km GSH values of other GSTs [25–29]. This result suggests that the GSH-binding site of BaGST2 and BaGST2 may be similar to those of other GSTs.

In addition to conjugation reactions, BaGST2 and BaGST3 have peroxidase activity. Peroxidase activity is of particular importance because in contrast to Se-dependent GSH-peroxidase activity, GST peroxidase is Se independent and may having a role in cellular antioxidant defense by reducing organic hydroperoxides. Sigma-class GSTs metabolize 4-hydroxynonenal which is a by-product of lipid peroxidation produced during the breakdown of long chain lipid hydroperoxides [32, 33]. In this context, the observed activity of BaGST2 and BaGST3 toward CuOOH indicates their role in antioxidant defense and metabolism of lipid peroxidation by-product in *B. alexandrina*. The specific activity found for BaGST2 and BaGST3 on CuOOH is comparable to that reported for *Fasciola hepatica* GST class sigma [34] but 100-fold lower if compared with GST class sigma from the insect *Phlebotomus argentipes* [33]. In addition to being inhibitor of GSTs, EA interacts with GSTs as a substrate for conjugation with GSH to yield an EA-GSH conjugate. BaGST2 and BaGST3 showed a comparatively high specific activity values with EA as that reported for the pi-class GSTs [35]. Compounds well-established as GST inhibitors were classified into three classes, determined by their binding site on the protein and mechanism of inhibition. The first class of inhibitors have structural analog to electrophilic substrates and competitively inhibits the binding of hydrophobic substrates to the H-site. The second are GSH conjugates, which occupy both the GSH binding site and at least part of the H-site and are competitive with respect to both GSH and hydrophobic substrate. The third class of inhibitors of compounds referred to as non-substrate ligands which are noncompetitive inhibitors of GSTs [36]. In the present investigation, hematin and CB were the most potent inhibitors for BaGST2 and BaGST3, with IC_50_ approximately of 0.5 μM, whereas in IC_50_ values of S-hexyl-GSH, S-butyl-GSH, and S-P-bromobenzyl-GSH, BSP are approximatly100-fold higher but remain within the range reported for other GSTs [35, 37]. The type of inhibition of CB is competitive for BaGST2 and BaGST3. However, kinetic inhibition studies, molecular modeling, and molecular dynamic simulations showed that the CB binding site overlaps both the G- and H-sites of *Schistosoma japonicum* (SjGST). CB interacts with SjGST by hydrophobic and polar interactions. Steric effects were also found [38]. It is worth mentioning that IC_50_ values of CB for BaGST2 and BaGST3 are 100-fold lower than that for SjGST. BSP (non-substrate anionic ligand) inhibition studies showed that BSP binds to the human GST class alpha in two independent sites, a high-affinity site and a low-affinity site. Binding of BSP to its high-affinity site does not inhibit the enzyme. The low-affinity-binding site(s) for BSP can simultaneously accommodates both BSP and the relatively small size electrophilic substrate, CDNB, and inhibits the enzyme non-competitively [39]. BSP also inhibits a 26-kDa *Schistosoma* GST from bovis/haematobium in a noncompetitive manner [40]. In the present investigation, BSP was found to be a competitive inhibitor for BaGST2 and mixed inhibitor for BaGST3. This could be due to the difference in H-site between sigma and alpha class GSTs.

The half-life times at 50 °C for BaGST2 and BaGST3 were 15 min and 25 min, respectively. The presence of a physiologically relevant concentration of GSH (5 mM) in the incubation buffer increased the stability of both BaGST2 and BaGST3 considerably to more than 180 min. Structurally, cytosolic GSTs function as dimers; each monomer is composed of a conserved thioredoxin domain containing the GSH binding site followed by a more variable α-helical domain. The fundamental theme in GST catalysis is the activation of GSH by the stabilization of the GSH thiolate [41]. Binding of GSH to the active site increases the interactions with the opposing subunit to 15 interactions, while only 8 interactions are found in the apoenzyme [42]. A similar result was reported for the increase in GST thermal stability in the presence of BSP and GSH [40].

Isoelectric focusing of BaGST3 indicated the presence of one band, while that of BaGST2 showed two bands. The presence of more than one band after electrofocusing of apparently homogeneous enzyme preparation of GSTs from different sources has been reported [10, 27]. Purified GSTs may display multiple bands on electrofocusing due to different reasons. These reasons include the resolution characteristics of the electrofocusing system, the existence of charged substituents on the protein (i.e., phosphate), and intramolecular sulfhydryl oxidation [43]. The theoretical isoelectric point for BaGST2 was 8.58, which is higher than that measured experimentally (two bands at pI 4.47 and 4.67). The difference between theoretical isoelectric point and the actual pI of a protein may be due to that some of the charged side chains are either buried or in salt bridges. The local environment of charged side chains can also affect their pKa. The higher theoretical pI could be due to the unidentified gaps in the predicted amino acid sequences.

In the present investigation, the pH optima for BaGST2, and BaGST3, were 9.5 and 6.0, respectively. Most of reported pH optima for GSTs are in the range between 6.5 and 8.5. A fewer GSTs have alkaline pH. BaGST2 exhibited alkaline pH optimum similar to that reported for GST purified from the insect *Musca domestica* [44].

The difference in the biochemical characteristic for BaGST2 and BaGST3 is not surprising even though the enzymes are very similar in structure. Two murine Pi class GSTs have the same number of amino acid sequences but different only in six amino acid residues. The activity of one isoenzyme toward a panel of electrophilic substrates, CDNB, EA, p-nitrobenzyl chloride, and CuOOH is 700-, 150-, 25-, and 9-fold lower than the other, respectively [45]. The differences in biochemical and immunological characteristics of BaGST2 and BaGST3 might be due to a few amino acid substitutions. Purified GST from the digestive gland of the squid *Ommastrephes sloani pacificus* revealed two polypeptide chains, one major and one minor band. These isolated polypeptides gave identical patterns of tryptic peptides after high-performance liquid chromatography separation [46].

According to Mannervik [29], positive cross-reactivity indicates membership of the same GST class. In the present investigation, anti-BaGST2 antibodies and anti-BaGST3 antibodies cross-react with the affinity-purified GSTs from *L. truncatula* and *P. acuta*. This means that members of the same class exist in different snail species. Surprisingly, anti-BaGST2 antibodies and anti-BaGST3 antibodies do not cross-react with the affinity-purified GSTs from *B. truncatus*, although *B. truncatus* and *B. alexandrina* snails are belonging to the same family (Planorbidae), whereas *L*. *truncatul*a and *P. acuta* are belonging to Lymnaeidae and Physidae, respectively.

As shown in Table 5, peptides no. 13 and no. 11 have the same position in the polypeptide chain. This could be due to a variety of reasons. The most probable reason is the presence of two hetero-subunits within BaGST2, each having different peptide. Some GST classes have similar members that can hybridize such that both homodimers and heterodimers occur [47]. Electrofocusing of BaGST2 confirms this probability with the presence of more than one bands. The probability of contamination with other proteins could be considered as another reason. However, this reason could be excluded because the peptide has a GST sequence.

The subunit MW of BaGST2 and BaGST3 was estimated to be approximately 23.6 kDa by SDS-PAGE. However, size-exclusion chromatography in Sephadex G-75 showed that the MW of BaGST3 was approximately 45 kDa; this indicates that this isoenzyme is a dimeric protein. The predicted amino acid sequences for BaGST2 are in agreement in length with sigma GST sequences [34]. Also, the predicted subunit MW of BaGST2 (22.6 kDa) is consistent with sigma-class GSTs possessing an average subunit MW of 23 kDa [48]. Moreover, this value is comparable to that measured by SDS-PAGE for freshwater snail GSTs [10, 26]. The theoretical calculation of subunit MW for *B. glabrata* GST7 and GST3, *B. truncates* GST1, *Haliotis discus discus* GST3, and *Haliotis rubra* GST2 is also in the range of 23 kDa.

Secondary structural analysis revealed the presence of 4β-sheets and 3α-helices in the N-terminal region and 6α-helices in the C-terminal region of BaGST2. The overall structure of BaGST2 at the N-terminal domain seems completely identical to the canonical GST N-terminal domain, has the typical thioredoxin-like fold with a βαβ-α-ββα motif, and similar to other cellular homeostasis and detoxification proteins such as GSH peroxidases and glutaredoxins [3]. BaGST2 N-terminal domain constitutes approximately one-third of the protein structure (5–82 amino acids). The β-β-α motif in the N-terminal domain, the G-site, is most conserved among the isoforms and provides the binding site for GSH by recognizing the γ-glutamyl fragment of GSH [49]. Within this site, a specific residue activates the GSH cysteinyl side chain through hydrogen bonding. In some classes (alpha, mu, pi, and sigma), this residue is a tyrosine while in some other (kappa and theta) is a serine or a cysteine [48]. BaGST2 has a conservative Tyr11 residue in the N-terminal G-site, which is a shared feature for sigma-class GSTs responsible for GSH stabilization. The G-site exclusively binds GSH and is highly conserved, while the H-site accepts more variability so to accommodate an extensive range of toxic electrophilic substances [2].

Crystal structural data from GSTs indicates that Pro-53 (Pro-55 in BaGST2) adopts the cis-configuration. Pro-53 is located in a β-turn that lines the base of the G-site and is important for the proper folding and maintenance of conformation of the G-site [50]. Moreover, BaGST2 can be clearly seen that nests in the sigma clade in the phylogenetic tree derived from different classes of GSTs. This structural features are similar to other sigma GST orthologs in *Baylisascaris schroederi*, *Hyriopsis cumingii*, and *Dugesia japonica* [48, 51, 52]. Proteomic and in silico analysis was used to detect the GST superfamily in cytosol extract from *Fasciola gigantica* [50], *Bulinus* globosus [53], and two bivalve species (*Mytilus galloprovincialis* and *Corbicula fluminea*) [54]. The results revealed the presence of sigma, mu, omega, and zeta class GSTs in *Fasciola gigantica*, an alpha-class GST was identified in *Bulinus globusus*, and two sigma-class GST subunits were identified in *Mytilus galloprovincialis* and *Corbicula fluminea*.

## Conclusions

We have purified and characterized two GSTs from *B. alexandrina* snails. Our study broadens the biochemical information on freshwater snail GSTs by demonstrating the role of BaGSTs in defense mechanisms against structurally different electrophilic compounds. BaGST2 and BaGST3 have Se-independent peroxidase activity, which indicates their role in cellular antioxidant defense by reducing organic hydroperoxides in *B. alexandrina*. A polypeptide chain of 206 amino acids was predicted. The primary structure of BaGST2 showed 65% sequence identity with *Biomphalaria glabrata* GST7. Sequence analysis indicates that BaGST2 is a GST-N-sigma-like with a thioredoxin-like superfamily. Phylogenetic tree confirms that BaGST2 belongs to the sigma class of GSTs superfamily.

## Supplementary Information

**Additional file 1: Supplementary Table S1**. Amino acids composition of BaGST2 and BaGST3 purified from *B. alexandrina* snails.

**Additional file 2: Supplementary Figure S2**. Multiple alignment of BaGST2 sequence with GST class sigma sequences from human, rat, and mouse. The alignment was created using Clustal Omega multiple sequence alignment program. (*) indicates identical residues in all sequences, while (**:**) indicates the highly positive residues and (**.**) for moderately positive ones. Unidentified gaps marked by hyphens are introduced for better alignment.

**Additional file 3: Supplementary Figure S3**. The secondary structure content of Ba GST2 was predicted by JPred4 (http://www.compbio.dundee.ac.uk/jpred4/index_up.html). The predicted BaGST2 sequence was submitted to PDB BLAST to predict the closely related homologs. The structure of the *Bombyx mori* GST sigma (PDB ID: 3vpq) was selected as template for homology modeling (Blast E-value1e-37). a-N-terminal domain and C-terminal domain, b- structure elements.

**Additional file 4: Supplementary Figure S4**. The details of dimer interface and domain interface residues in both the N-terminal and the C- terminal domain of BaGST2. Conserved domains were identified by the tool of the CD-search based on the conserved domain database (CDD) (https://www.ncbi.nlm.nih.gov/Structure/cdd/wrpsb.cgi).

## Data Availability

The authors declare that all generated and analyzed data have been included in the article.

## Abbreviations

*B. alexandrina*: *Biomphalaria alexandrina*
BaGST: *Biomphalaria alexandrina* GST
*B. glabrata*: *Biomphalaria glabrata*
BSP: Bromosulfophthalein
CDNB: 1-Chloro-2,4-dinitrobenzene
DCNB: 1,2-Dichloro-4-nitrobenzene
CB: Cibacron Blue
CuOOH: Cumene hydroperoxide
ELISA: Enzyme-linked immunosorbent assay
EA: Ethacrynic acid
EDTA: Ethylenediaminetetraacetic acid
GSH: Glutathione
GSTs: Glutathione transferases
NM: Number of missed cleavage
ND: No detected activity
PAGE: Polyacrylamide gel electrophoresis
HPLC: Reversed-phase liquid chromatography
