# Supplementary material for: Biochemical characterization and peptide mass fingerprinting of two glutathione transferases from Biomphalaria alexandrina snails (Gastropoda: Planorbidae)
Source: J Genet Eng Biotechnol. 2022 Jul 6;20:99. doi: 10.1186/s43141-022-00372-x (PMC9259769; doi:10.1186/s43141-022-00372-x)
Supplement: Supplementary file 1 — Additional file 1: Supplementary Table S1. Amino acids composition of BaGST2 and BaGST3 purified from B. alexandrina snails. [file 43141_2022_372_MOESM1_ESM.docx]

Table 1: Amino acid composition of *B. alexandrina* GST2 and GST3

| Component | GST2  (number of amino acids/subunit) | GST3  (number of amino acids/subunit) |
| --- | --- | --- |
| Asp and Asn  Thr  Ser  Glu and Gln  Pro  Gly  Ala  Val  Met  Ile  Leu  Tyr  Phe  His  Lys  Arg | 21.50  10.69  12.62  23.97  8.35  16.95  20.44  15.60  0.98  12.05  19.19  7.66  12.05  3.90  16.34  10.17 | 22.63  11.93  13.04  24.19  8.25  20.20  21.29  14.79  2.44  12.45  17.25  7.33  11.26  3.59  14.29  10.50 |
