# Supplementary material for: Biochemical characterization and peptide mass fingerprinting of two glutathione transferases from Biomphalaria alexandrina snails (Gastropoda: Planorbidae)
Source: J Genet Eng Biotechnol. 2022 Jul 6;20:99. doi: 10.1186/s43141-022-00372-x (PMC9259769; doi:10.1186/s43141-022-00372-x)
Supplement: Supplementary file 2 — Additional file 2: Supplementary Figure S2. Multiple alignment of BaGST2 sequence with GST class sigma sequences from human, rat, and mouse. The alignment was created using Clustal Omega multiple sequence alignment program. (*) indicates identical residues in all sequences, while (:) indicates the highly positive residues and (.) for moderately positive ones. Unidentified gaps marked by hyphens are introduced for better alignment. [file 43141_2022_372_MOESM2_ESM.docx]

**Supplementary Figure S2**

**BaGST2 MAEAKNVKVLYFDVTGLGEILRLLLKFAGKEYEDVRFSFEEWPKXKPTTPFGQMPVLEVD 60**

**sp|O60760|HPGDS_HUMAN ---MPNYKLTYFNMRGRAEIIRYIFAYLDIQYEDHRIEQADWPEIKSTLPFGKIPILEVD 57**

**sp|O35543|HPGDS_RAT ---MPNYKLLYFNMRGRAEIIRYIFAYLDIKYEDHRIEQADWPKIKPTLPFGKIPVLEVE 57**

**sp|Q9JHF7|HPGDS_MOUSE ---MPNYKLLYFNMRGRAEIIRYIFAYLDIKYEDHRIEQADWPKIKPTLPFGKIPVLEVE 57**

*** *: **:: * .**:* :: : . :*** *:. :**: * * ***::*:***:**

**BaGST2 GKKKAQSIALAAFLAREFKXXGKDDLEALQVDATVDTIHDLRAKRFKSFRESDPVKkEAI 120**

**sp|O60760|HPGDS_HUMAN GLTLHQSLAIARYLTKNTDLAGNTEMEQCHVDAIVDTLDDFMSCF--PWAEKKQDVKEQM 115**

**sp|O35543|HPGDS_RAT GLTLHQSLAIARYLTKNTDLAGKTELEQCQVDAVVDTLDDFMSLF--PWAEENQDLKERT 115**

**sp|Q9JHF7|HPGDS_MOUSE GLTIHQSLAIARYLTKNTDLAGKTALEQCQADAVVDTLDDFMSLF--PWAEKDQDLKERM 115**

*** . **:*:* :*::: . *: :* :.** ***:.*: : : *.. ****

**BaGST2 VTEVKDVFLPKFMGFFESLLKKNGSTGLFVGKKLTWGDFVFAGIYAY-LKAAFEAIDNFP 179**

**sp|O60760|HPGDS_HUMAN FNELLTYNAPHL---MQDLDTYLGGREWLIGNSVTWADFYWEICSTTLLVFKPDLLDNHP 172**

**sp|O35543|HPGDS_RAT FNDLLTRQAPHL---LKDLDTYLGDKEWFIGNYVTWADFYWDICSTTLLVLKPDLLGIYP 172**

**sp|Q9JHF7|HPGDS_MOUSE FNELLTHQAPRL---LKDLDTYLGDKEWFIGNYVTWADFYWDICSTTLLVLKPGLLDIYP 172**

**..:: *:: ::.* . *. ::*: :**.** : : * :. .***

**BaGST2 LVKKLVDTVGDNERIKKWIETRPASKF 206**

**sp|O60760|HPGDS_HUMAN RLVTLRKKVQAIPAVANWIKRRPQTKL 199**

**sp|O35543|HPGDS_RAT RLVSLRNKVQAIPAISAWILKRPQTKL 199**

**sp|Q9JHF7|HPGDS_MOUSE KLVSLRNKVQAIPAISAWILKRPQTKL 199**

**: .* ..* : ** ** :*:**
