# Supplementary material for: Biochemical characterization and peptide mass fingerprinting of two glutathione transferases from Biomphalaria alexandrina snails (Gastropoda: Planorbidae)
Source: J Genet Eng Biotechnol. 2022 Jul 6;20:99. doi: 10.1186/s43141-022-00372-x (PMC9259769; doi:10.1186/s43141-022-00372-x)
Supplement: Supplementary file 3 — Additional file 3: Supplementary Figure S3. The secondary structure content of Ba GST2 was predicted by JPred4 (http://www.compbio.dundee.ac.uk/jpred4/index_up.html). The predicted BaGST2 sequence was submitted to PDB BLAST to predict the closely related homologs. The structure of the Bombyx mori GST sigma (PDB ID: 3vpq) was selected as template for homology modeling (Blast E-value1e-37). a-N-terminal domain and C-terminal domain, b- structure elements. [file 43141_2022_372_MOESM3_ESM.docx]

**Supplementary Figure S3a (N-terminal domain and C-terminal domain)**
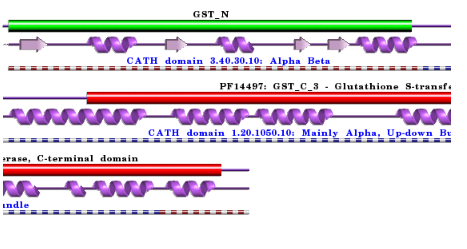


**Supplementary Figure b (structure elements)**


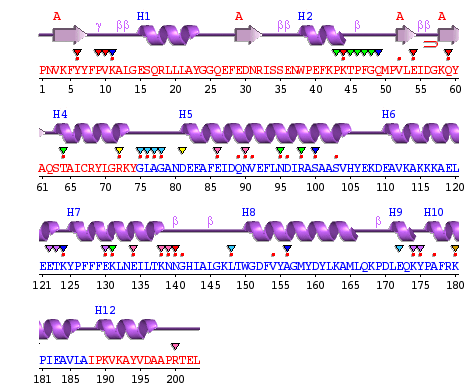


Supplementary Figure S3. The secondary structure content was predicted by JPred4 (<http://www.compbio.dundee.ac.uk/jpred4/index_up.html>). The BaGST2 sequence was submitted to PDB BLAST to predict the closely related homologs. The structure of the Bombyx mori GST sigma (PDB ID: [3vpq](http://www.ebi.ac.uk/pdbsum/3vpq)) was selected as template for homology modeling (Blast E-value1e-37).

| The secondary structure elements containing: |  |
| --- | --- |
| \|  \|  \| 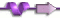 \| \|  \| Helices labelled H1, H2, ... and strands by their sheets A, ... \|  \| \| --- \| --- \| --- \| --- \| --- \| --- \| --- \| \|  \| \| Helix \| Strand \|  \| \| \| |  |
|  | |
| \| Motifs: \|  \| \|  \| 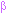 \|  \| beta turn, \|  \| 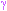 \|  \| gamma turn, and \|  \| 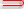 \|  \| beta hairpin \| \| --- \| --- \| --- \| --- \| --- \| --- \| --- \| --- \| --- \| --- \| --- \| --- \| \| \| --- \| --- \| --- \| --- \| --- \| --- \| --- \| --- \| --- \| --- \| --- \| --- \| --- \| --- \| --- \| |  |
