# Supplementary material for: Biochemical characterization and peptide mass fingerprinting of two glutathione transferases from Biomphalaria alexandrina snails (Gastropoda: Planorbidae)
Source: J Genet Eng Biotechnol. 2022 Jul 6;20:99. doi: 10.1186/s43141-022-00372-x (PMC9259769; doi:10.1186/s43141-022-00372-x)
Supplement: Supplementary file 4 — Additional file 4: Supplementary Figure S4. The details of dimer interface and domain interface residues in both the N-terminal and the C- terminal domain of BaGST2. Conserved domains were identified by the tool of the CD-search based on the conserved domain database (CDD) (https://www.ncbi.nlm.nih.gov/Structure/cdd/wrpsb.cgi). [file 43141_2022_372_MOESM4_ESM.docx]

**
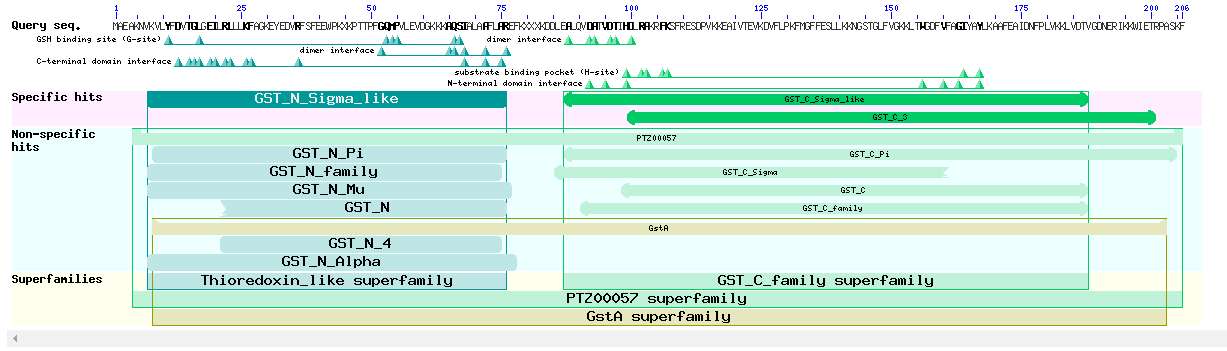
**

**Conserved Protein Domain Family *GST_N_Sigma_like***

**GSH binding site (G-site) [chemical binding site] (Feature 1**)

Feature 1 # # ### ##

query 7 VKVLYFDVTGLGEILRLLLKFAGKEYEDVRFS.[13].GQMPVLEVD GKKKAQSIALAAFLAR 76 BaGST2

[1IYI_D](https://www.ncbi.nlm.nih.gov/protein/1IYI_D?report=GenPept) 3 YKLTYFNMRGRAEIIRYIFAYLDIQYEDHRIE.[13].GKIPILEVD GLTLHQSLAIARYLTK 72 [human](https://www.ncbi.nlm.nih.gov/Taxonomy/Browser/wwwtax.cgi?id=9606)

[1Q4J_B](https://www.ncbi.nlm.nih.gov/protein/1Q4J_B?report=GenPept) 5 IVLYYFDARGKAELIRLIFAYLGIEYTDKRFG.[20].EQVPILQIG DLILAQSQAIVRYLSK 81 [m. p. P. f.](https://www.ncbi.nlm.nih.gov/Taxonomy/Browser/wwwtax.cgi?id=5833)

[P27012](https://www.ncbi.nlm.nih.gov/protein/134283?report=GenPept) 4 YTLHYFNHRGRAEICRMLFAAAGVQYNDRRIE.[13].SMMPMLELD.[1].KIQIPQSMAMARYLAR 74 [Octopus dofleini](https://www.ncbi.nlm.nih.gov/Taxonomy/Browser/wwwtax.cgi?id=6644)

[AAA97540](https://www.ncbi.nlm.nih.gov/protein/625079?report=GenPept) 4 YTLYYFNGRGRAEICRMIFAAAAIQYNDKRIE.[13].HMLPILEID.[1].DTQVPQSMAISRYLAR 74 [Loligo opalescens](https://www.ncbi.nlm.nih.gov/Taxonomy/Browser/wwwtax.cgi?id=31211)

**dimer interface [polypeptide binding site] (Feature 2)**

Feature 2 # ## # # #

query 7 VKVLYFDVTGLGEILRLLLKFAGKEYEDVRFS.[13].GQMPVLEVD GKKKAQSIALAAFLAR 76 BaGST2

[1IYI_D](https://www.ncbi.nlm.nih.gov/protein/1IYI_D?report=GenPept) 3 YKLTYFNMRGRAEIIRYIFAYLDIQYEDHRIE.[13].GKIPILEVD GLTLHQSLAIARYLTK 72 [human](https://www.ncbi.nlm.nih.gov/Taxonomy/Browser/wwwtax.cgi?id=9606)

[1Q4J_B](https://www.ncbi.nlm.nih.gov/protein/1Q4J_B?report=GenPept) 5 IVLYYFDARGKAELIRLIFAYLGIEYTDKRFG.[20].EQVPILQIG DLILAQSQAIVRYLSK 81 m. p. P. f

[P27012](https://www.ncbi.nlm.nih.gov/protein/134283?report=GenPept) 4 YTLHYFNHRGRAEICRMLFAAAGVQYNDRRIE.[13].SMMPMLELD.[1].KIQIPQSMAMARYLAR 74 [Octopus dofleini](https://www.ncbi.nlm.nih.gov/Taxonomy/Browser/wwwtax.cgi?id=6644)

[AAA97540](https://www.ncbi.nlm.nih.gov/protein/625079?report=GenPept) 4 YTLYYFNGRGRAEICRMIFAAAAIQYNDKRIE.[13].HMLPILEID.[1].DTQVPQSMAISRYLAR 74 [Loligo opalescens](https://www.ncbi.nlm.nih.gov/Taxonomy/Browser/wwwtax.cgi?id=31211)

**C-terminal domain interface [polypeptide binding site] (Feature 3)**

Feature 3 # ### ## ## ## # # # #

query 7 VKVLYFDVTGLGEILRLLLKFAGKEYEDVRFS.[13].GQMPVLEVD GKKKAQSIALAAFLAR 76 BaGST2

[1IYI_D](https://www.ncbi.nlm.nih.gov/protein/1IYI_D?report=GenPept) 3 YKLTYFNMRGRAEIIRYIFAYLDIQYEDHRIE.[13].GKIPILEVD GLTLHQSLAIARYLTK 72 [human](https://www.ncbi.nlm.nih.gov/Taxonomy/Browser/wwwtax.cgi?id=9606)

[1Q4J_B](https://www.ncbi.nlm.nih.gov/protein/1Q4J_B?report=GenPept) 5 IVLYYFDARGKAELIRLIFAYLGIEYTDKRFG.[20].EQVPILQIG DLILAQSQAIVRYLSK 81 m. p. P. f

[P27012](https://www.ncbi.nlm.nih.gov/protein/134283?report=GenPept) 4 YTLHYFNHRGRAEICRMLFAAAGVQYNDRRIE.[13].SMMPMLELD.[1].KIQIPQSMAMARYLAR 74 [Octopus dofleini](https://www.ncbi.nlm.nih.gov/Taxonomy/Browser/wwwtax.cgi?id=6644)

[AAA97540](https://www.ncbi.nlm.nih.gov/protein/625079?report=GenPept) 4 YTLYYFNGRGRAEICRMIFAAAAIQYNDKRIE.[13].HMLPILEID.[1].DTQVPQSMAISRYLAR 74 [Loligo opalescens](https://www.ncbi.nlm.nih.gov/Taxonomy/Browser/wwwtax.cgi?id=31211)

**Conserved Protein Domain Family *GST_C_Sigma_like***

**dimer interface [polypeptide binding site] (Feature 1)**

| Feature 1 # ## ## #  query 87 EALQVDATVDTIHDLRAKRFK.[13].VTE VKDV.[1].LPKFMGFFESLL.[6].GLFVG.[1].KLTWGDFVF 161 BaGST2  [1V40_D](https://www.ncbi.nlm.nih.gov/protein/1V40_D?report=GenPept) 83 EQCHVDAIVDTLDDFMSCFPW.[10].MFN ELLT.[2].APHLMQDLDTYL.[3].EWLIG.[1].SVTWADFYW 152 [human](https://www.ncbi.nlm.nih.gov/Taxonomy/Browser/wwwtax.cgi?id=9606)  [AAV31410](https://www.ncbi.nlm.nih.gov/protein/54287926?report=GenPept) 84 ESLLIDIAVDNIHDLRLAIAG.[12].KYA.[1].LINE.[1].IPFYMDKFEKFV.[4].GYFVN.[1].KLSWADLFF 156 b.c. a.)  [CAA71620](https://www.ncbi.nlm.nih.gov/protein/1805650?report=GenPept) 84 EEAQVDSIFDQFKDFMAELRP.[16].LKE VAVP.[1].RDKHLPLLEKFL.[5].EYMVG.[1].SVTWADLVI 160 [p.r.w.](https://www.ncbi.nlm.nih.gov/Taxonomy/Browser/wwwtax.cgi?id=6253)  [CAB61107](https://www.ncbi.nlm.nih.gov/protein/6434557?report=GenPept) 86 EQAWADAIVDQFKDFMGAFRQ.[17].SSE VAIP.[1].RDSYFKILNGLL.[5].GFLVG.[1].GLTFADIVV 163 [nematode](https://www.ncbi.nlm.nih.gov/Taxonomy/Browser/wwwtax.cgi?id=6239) |
| --- |
| Feature 1  query 162 AGIYAYLK.[10].LVKKLVDTV 188 BaGST2  [1V40_D](https://www.ncbi.nlm.nih.gov/protein/1V40_D?report=GenPept) 153 EICSTTLL.[11].RLVTLRKKV 180 [human](https://www.ncbi.nlm.nih.gov/Taxonomy/Browser/wwwtax.cgi?id=9606)  [AAV31410](https://www.ncbi.nlm.nih.gov/protein/54287926?report=GenPept) 157 VAVLDYLN.[12].NLKALKEKV 185 [brown citrus aphid](https://www.ncbi.nlm.nih.gov/Taxonomy/Browser/wwwtax.cgi?id=223852)  [CAA71620](https://www.ncbi.nlm.nih.gov/protein/1805650?report=GenPept) 161 TDSLASWE.[11].QLKKYIEHV 188 [pig roundworm](https://www.ncbi.nlm.nih.gov/Taxonomy/Browser/wwwtax.cgi?id=6253)  [CAB61107](https://www.ncbi.nlm.nih.gov/protein/6434557?report=GenPept) 164 VENLTTLE.[11].KLSALREKV 191 [nematode](https://www.ncbi.nlm.nih.gov/Taxonomy/Browser/wwwtax.cgi?id=6239) |

**Substrate binding pocket (H-site) [chemical binding site] (Feature 2)**

| Feature 2 # ## ##  query 87 EALQVDATVDTIHDLRAKRFK.[13].VTE VKDV.[1].LPKFMGFFESLL.[6].GLFVG.[1].KLTWGDFVF 161 BaGST2  [1V40_D](https://www.ncbi.nlm.nih.gov/protein/1V40_D?report=GenPept) 83 EQCHVDAIVDTLDDFMSCFPW.[10].MFN ELLT.[2].APHLMQDLDTYL.[3].EWLIG.[1].SVTWADFYW 152 [human](https://www.ncbi.nlm.nih.gov/Taxonomy/Browser/wwwtax.cgi?id=9606)  [AAV31410](https://www.ncbi.nlm.nih.gov/protein/54287926?report=GenPept) 84 ESLLIDIAVDNIHDLRLAIAG.[12].KYA.[1].LINE.[1].IPFYMDKFEKFV.[4].GYFVN.[1].KLSWADLFF 156 [b.c.a](https://www.ncbi.nlm.nih.gov/Taxonomy/Browser/wwwtax.cgi?id=223852)  [CAA71620](https://www.ncbi.nlm.nih.gov/protein/1805650?report=GenPept) 84 EEAQVDSIFDQFKDFMAELRP.[16].LKE VAVP.[1].RDKHLPLLEKFL.[5].EYMVG.[1].SVTWADLVI 160 P.r.w.  [CAB61107](https://www.ncbi.nlm.nih.gov/protein/6434557?report=GenPept) 86 EQAWADAIVDQFKDFMGAFRQ.[17].SSE VAIP.[1].RDSYFKILNGLL.[5].GFLVG.[1].GLTFADIVV 163 [nematode](https://www.ncbi.nlm.nih.gov/Taxonomy/Browser/wwwtax.cgi?id=6239) |
| --- |
| Feature 2 # #  query 162 AGIYAYLK.[10].LVKKLVDTV 188 BaGST2  [1V40_D](https://www.ncbi.nlm.nih.gov/protein/1V40_D?report=GenPept) 153 EICSTTLL.[11].RLVTLRKKV 180 [human](https://www.ncbi.nlm.nih.gov/Taxonomy/Browser/wwwtax.cgi?id=9606)  [AAV31410](https://www.ncbi.nlm.nih.gov/protein/54287926?report=GenPept) 157 VAVLDYLN.[12].NLKALKEKV 185 [brown citrus aphid](https://www.ncbi.nlm.nih.gov/Taxonomy/Browser/wwwtax.cgi?id=223852)  [CAA71620](https://www.ncbi.nlm.nih.gov/protein/1805650?report=GenPept) 161 TDSLASWE.[11].QLKKYIEHV 188 [pig roundworm](https://www.ncbi.nlm.nih.gov/Taxonomy/Browser/wwwtax.cgi?id=6253)  [CAB61107](https://www.ncbi.nlm.nih.gov/protein/6434557?report=GenPept) 164 VENLTTLE.[11].KLSALREKV 191 [nematode](https://www.ncbi.nlm.nih.gov/Taxonomy/Browser/wwwtax.cgi?id=6239) |

**N-terminal domain interface [polypeptide binding site]( Feature 3)**

| Feature 3 # # # # #  query 87 EALQVDATVDTIHDLRAKRFK.[13].VTE VKDV.[1].LPKFMGFFESLL.[6].GLFVG.[1].KLTWGDFVF 161 BaGST2  [1V40_D](https://www.ncbi.nlm.nih.gov/protein/1V40_D?report=GenPept) 83 EQCHVDAIVDTLDDFMSCFPW.[10].MFN ELLT.[2].APHLMQDLDTYL.[3].EWLIG.[1].SVTWADFYW 152 [human](https://www.ncbi.nlm.nih.gov/Taxonomy/Browser/wwwtax.cgi?id=9606)  [AAV31410](https://www.ncbi.nlm.nih.gov/protein/54287926?report=GenPept) 84 ESLLIDIAVDNIHDLRLAIAG.[12].KYA.[1].LINE.[1].IPFYMDKFEKFV.[4].GYFVN.[1].KLSWADLFF 156 [b.c. a.](https://www.ncbi.nlm.nih.gov/Taxonomy/Browser/wwwtax.cgi?id=223852)  [CAA71620](https://www.ncbi.nlm.nih.gov/protein/1805650?report=GenPept) 84 EEAQVDSIFDQFKDFMAELRP.[16].LKE VAVP.[1].RDKHLPLLEKFL.[5].EYMVG.[1].SVTWADLVI 160 P.r.worm [CAB61107](https://www.ncbi.nlm.nih.gov/protein/6434557?report=GenPept) 86 EQAWADAIVDQFKDFMGAFRQ.[17].SSE VAIP.[1].RDSYFKILNGLL.[5].GFLVG.[1].GLTFADIVV 163 [nematode](https://www.ncbi.nlm.nih.gov/Taxonomy/Browser/wwwtax.cgi?id=6239) |
| --- |
| Feature 3 # #  query 162 AGIYAYLK.[10].LVKKLVDTV 188 BaGST2  [1V40_D](https://www.ncbi.nlm.nih.gov/protein/1V40_D?report=GenPept) 153 EICSTTLL.[11].RLVTLRKKV 180 [human](https://www.ncbi.nlm.nih.gov/Taxonomy/Browser/wwwtax.cgi?id=9606)  [AAV31410](https://www.ncbi.nlm.nih.gov/protein/54287926?report=GenPept) 157 VAVLDYLN.[12].NLKALKEKV 185 [brown citrus aphid](https://www.ncbi.nlm.nih.gov/Taxonomy/Browser/wwwtax.cgi?id=223852)  [CAA71620](https://www.ncbi.nlm.nih.gov/protein/1805650?report=GenPept) 161 TDSLASWE.[11].QLKKYIEHV 188 [pig roundworm](https://www.ncbi.nlm.nih.gov/Taxonomy/Browser/wwwtax.cgi?id=6253)  [CAB61107](https://www.ncbi.nlm.nih.gov/protein/6434557?report=GenPept) 164 VENLTTLE.[11].KLSALREKV 191 [nematode](https://www.ncbi.nlm.nih.gov/Taxonomy/Browser/wwwtax.cgi?id=6239) |

[malaria parasite P. falciparum](https://www.ncbi.nlm.nih.gov/Taxonomy/Browser/wwwtax.cgi?id=5833) (m.p. falciparum)

[pig roundworm](https://www.ncbi.nlm.nih.gov/Taxonomy/Browser/wwwtax.cgi?id=6253) (P.r.w.)

[brown citrus aphid](https://www.ncbi.nlm.nih.gov/Taxonomy/Browser/wwwtax.cgi?id=223852) (b.c.a.)

[m. p. P. falciparum](https://www.ncbi.nlm.nih.gov/Taxonomy/Browser/wwwtax.cgi?id=5833)

Conserved domains were identified by the tool of the CD-search based on the Conserved Domain Database (CDD) (https://www.ncbi.nlm.nih.gov/ Structure/cdd/wrpsb.cgi)
